# Combination of platelet-rich plasma and bone marrow mesenchymal stem cells enhances tendon–bone healing in a rabbit model of anterior cruciate ligament reconstruction

**DOI:** 10.1186/s13018-016-0433-7

**Published:** 2016-09-07

**Authors:** Chong Teng, Chenhe Zhou, Danfeng Xu, Fanggang Bi

**Affiliations:** 1 Department of Orthopaedic Surgery, The Fourth Affiliated Hospital, School of Medicine, Zhejiang University, Yiwu, 322000 China; 2 Department of Orthopaedic Surgery, The Second Affiliated Hospital, School of Medicine, Zhejiang University, Hangzhou, 310009 China; 3 Department of Orthopaedic Surgery, The First Affiliated Hospital of Zhengzhou University, Zhengzhou, 450001 China

**Keywords:** Platelet-rich plasma, Bone marrow mesenchymal stem cells, Anterior cruciate ligament, Reconstruction, Tendon–bone healing

## Abstract

**Background:**

The objective of this study was to investigate the potency of platelet-rich plasma (PRP) combined with bone marrow mesenchymal stem cells (BMSCs) to promote tendon–bone healing in a rabbit model.

**Methods:**

In the in vitro study, the effects of PRP on osteogenic induction of BMSCs were analysed. Later, PRP with or without BMSCs was used in the rabbit model of anterior cruciate ligament reconstruction. Specimens were harvested 8 weeks postoperatively to evaluate tendon–bone healing by histology, radiology, and biomechanical testing.

**Results:**

The in vitro study revealed that collagen I, osteocalcin, and osteopontin expression was higher in BMSCs co-cultured with PRP for 14 days. The in vivo study revealed a more mature tendon–bone interface using light microscopy, a more newly formed bone at the bone tunnel walls detected by micro-computed tomography, and a significantly higher failure load as assessed by biomechanical testing in the BMSC + PRP group than in the control and PRP groups.

**Conclusions:**

These results indicate that the combination of PRP and BMSCs promotes tendon–bone healing and has potential for clinical use.

**Electronic supplementary material:**

The online version of this article (doi:10.1186/s13018-016-0433-7) contains supplementary material, which is available to authorized users.

## Background

Anterior cruciate ligament (ACL) injuries are common, frequently lead to knee disability, and have low intrinsic regenerative ability [1]. ACL reconstruction is the gold standard because of its positive outcomes. Hamstring tendon grafts have been widely used due to the lower donor-site morbidity compared to bone–patellar tendon–bone (B-PT-B) grafts [2]. However, tendon–bone healing between a hamstring tendon graft and the created bone tunnels is associated with significantly slower bone healing compared with the B-PT-B graft [3]. Therefore, means for improving tendon–bone healing after reconstruction have become a major research focus in sports medicine [4].

Platelet-rich plasma (PRP), an autologous enriched source of various growth factors, such as platelet-derived growth factor (PDGF), platelet-derived epidermal growth factor (PDEGF), platelet-derived angiogenesis factor, transforming growth factor (TGF), insulin-like growth factor, and vascular endothelial growth factor (VEGF), has been widely used during trauma and orthopaedic surgery [5, 6]. PRP can clinically accelerate the healing of hard and soft tissues after maxillofacial, plastic, dermatologic, dental, and orthopaedic surgeries [7]. However, existing preclinical and clinical evidence regarding PRP use in ACL surgery failed to demonstrate a clear benefit [8]. Bone marrow mesenchymal stem cells (BMSCs) have attracted much interest and have become ideal seed cells for tissue engineering because of their multipotentiality and self-renewal potential, as well as their possible suitability for clinical use [9]. BMSCs have outstanding potential to promote tendon regeneration [10], and their osteogenic differentiation potential in vitro and bone formation capability on biodegradable scaffolds in vivo have been characterised in several studies [11, 12]. Many studies have shown that host cells containing BMSCs from surrounding bone tunnel bone marrow contribute to tendon–bone healing [13, 14]. However, BMSCs in bone marrow are rare, with only one BMSC detected in 1 × 10^5^ bone marrow mononuclear cells. PRP, as a storage vehicle for growth factors such as PDGF, TGF-β, PDEGF, VEGF, and platelet factor-4, enhances osteogenic differentiation of BMSCs [15]. Many studies have shown that factors that positively affect bone formation and fracture healing also positively affect tendon–bone healing [16].

In the present study, we seeded PRP and BMSCs at the tendon–bone interface using fibrin glue to explore whether tendon–bone healing is promoted by providing BMSCs and an osteoinductive factor.

## Methods

### Preparation of PRP

Autologous PRP was prepared as described previously [17]. Briefly, 10 mL of whole blood was drawn from the marginal auricular vein using an 18-gauge catheter. The blood was injected into a sterile centrifuge tube containing 1.5 mL of sodium citrate. The mixture was centrifuged at 1200×*g* for 10 min to separate the plasma from the red blood cells. The plasma was centrifuged again at 2500×*g* at 4 °C for 20 min, and the precipitated platelets (1 mL) were collected.

### Isolation and culture of BMSCs

BMSCs were generated from bone marrow aspirates of New Zealand White rabbits (age, 12 weeks; weight, 2.5 ± 0.2 kg), as described previously [18]. Mononuclear cells were collected after centrifugation in Ficoll–Hypaque gradient (Sigma Co., St. Louis, MO, USA) and resuspended in Dulbecco’s modified Eagle medium (DMEM) containing 10 % fetal bovine serum (FBS; Gibco, Grand Island, NY, USA). After a 72-h incubation at 37 °C in 5 % CO_2_, the non-adherent cells were removed by changing the culture medium. Adherent cells were subcultured when they reached 70–80 % confluence. A homogenous BMSC population was obtained after 2 weeks of culture, and the third passage was harvested for further use. The passage 3 cells were identified by detecting surface antigen marker expression profiles using flow cytometry. The osteogenic, chondrogenic, and adipogenic differentiation abilities of the cells were determined using inducing media for 3 weeks. Alizarin red, oil red O, and alcian blue staining were performed.

### Induction of osteogenic gene expression by PRP

Third passage BMSCs were harvested by trypsinisation and centrifugation. After culturing for 24 h, the original culture medium was removed. The BMSCs were washed three times with PBS and incubated in DMEM with 10 % PRP. In the control group, BMSCs were incubated in DMEM with 10 % FBS. Total RNA was extracted from cells cultured for 3, 7, and 14 days using TRIzol reagent (Invitrogen, Carlsbad, CA, USA). RNA concentration was determined with the NanoDrop spectrophotometer (NanoDrop Technologies, Wilmington, DE, USA), and 200 ng of RNA was used to synthesise complementary DNA (cDNA) using the iScript cDNA synthesis kit (Bio-Rad Laboratories, Hercules, CA, USA). The Stratagene M×3000P system (Stratagene, La Jolla, CA, USA) was used to perform and monitor the reactions. The QuantiTect SYBR Green PCR kit (Qiagen, Valencia, CA, USA) was used to quantify transcription levels of osteogenic genes, including collagen I, osteocalcin, and osteopontin. The glyceraldehyde-3-phosphate dehydrogenase gene was amplified in parallel with the target genes. The primer sequences are listed in Table 1.

**Table 1:** Primers of collagen I, OCN, and OPN used in RT-PCR in this study

Gene		Primer sequence
GAPDH	Forward	5′-ATGGGGAAGGTGAAGGTCG-3′
	Reverse	5′-TAAAAGCAGCCCTGGTGACC-3′
Collagen I	Forward	5′-GGTTTGTTGAAGAGGCTG-3′
	Reverse	5′-GATGGCCTGAAGCTCAA-3′
Osteocalcin	Forward	5′-CCGGGAGGAGATCTGTGAAA-3′
	Reverse	5′-CTGCCTTCTTCCACAATTTTATCC-3′
Osteopontin	Forward	5′-GCCAGTTGCAGCCTTCTCA-3′
	Reverse	5′-GCCATGCCCAAGAGACAAAA-3′

### ACL reconstruction model in rabbits

**Fig. 1:**
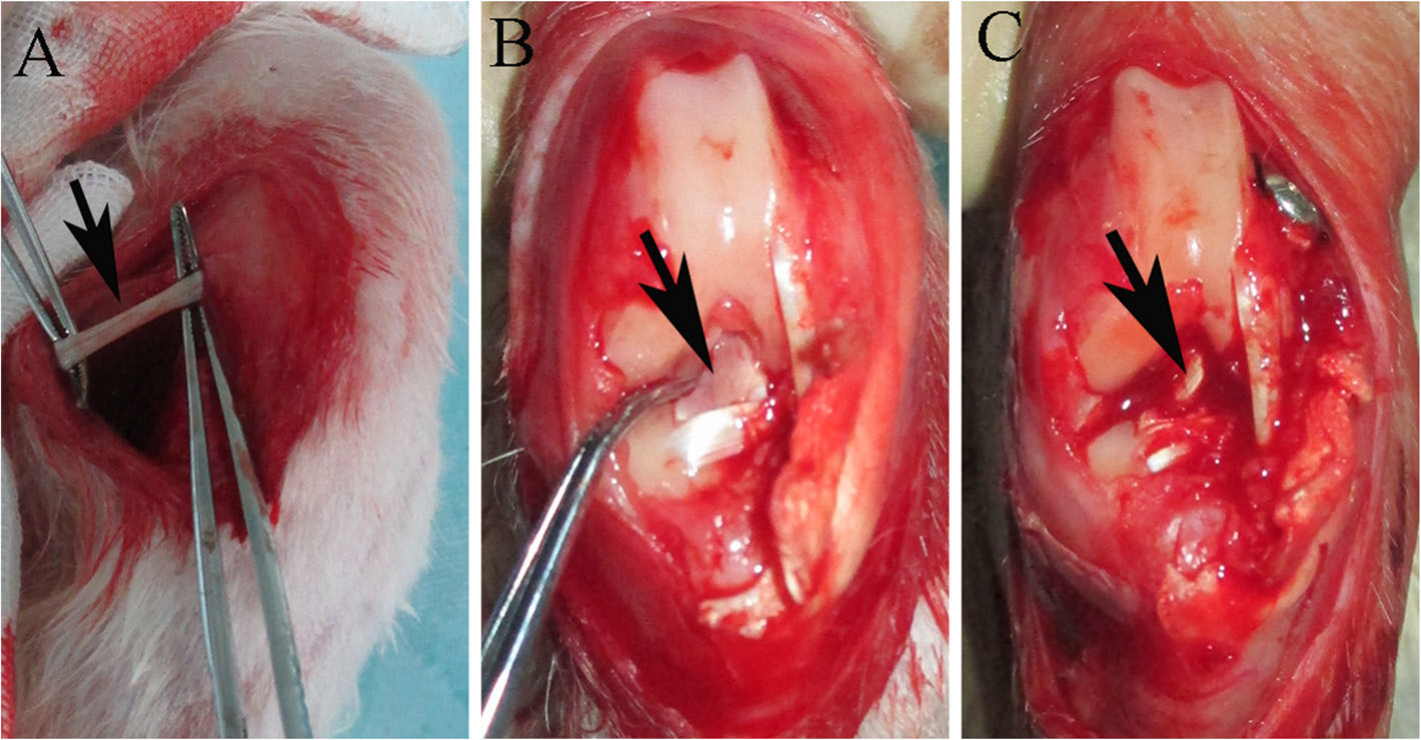
**a** Gross observations of the semitendinosus tendon (*black arrow*) and **b** native anterior cruciate ligament (ACL) (*black arrow*). **c** Macroscopic view of ACL reconstruction (*arrow* points to autologous semitendinosus tendon graft)

Animal experiments were approved by the Zhejiang University Ethics Committee. A total of 30 New Zealand White rabbits (age, 12 weeks, and weight, 2.5 ± 0.2 kg) were used in this study. All rabbits underwent ACL reconstruction in the left hind leg after intravenous anaesthesia with 30 mg/kg body weight pentobarbital sodium solution (Dawen Biotech, Seoul, Korea). After shaving and disinfecting the left hind leg, a lateral parapatellar incision was made to expose the knee joint. After the native ACL was excised, the tibial and femoral bone tunnels were created with a 2.5-mm-diameter drill (Fig. 1b). The ipsilateral semitendinosus tendon was harvested to reconstruct the ACL (Fig. 1a). The rabbits were divided randomly into three groups. In the first group, a normal hamstring tendon was used for ACL reconstruction (control group). Rabbits from the second group (PRP group) received hamstring tendons wrapped with 0.1 mL PRP immobilised in 0.1 mL fibrin glue (TISSEEL kit; Baxter AG, Vienna, Austria) for ACL reconstruction. In the third group (BMSC + PRP group), 1 × 10^7^ BMSCs were immobilised in 0.2 mL PRP and 0.1 mL fibrin glue and used for ACL reconstruction. The PRP concentration and number of BMSCs were determined by flow cytometry. Grafts for the PRP and BMSC + PRP groups were wrapped with glue immediately before insertion into the bone tunnels. Both ends of the graft were fixed by sutures tied over screws in the femur and tibia (Fig. 1c). Animals were allowed to move freely postoperatively. All rabbits were sacrificed with a lethal injection of pentobarbital 8 weeks postoperatively for assessment; half of the specimens in each group (*n* = 5/group) were used for the histological assessment and the other half (*n* = 5/group) were used for radiological and biomechanical assessments.

### Histology

The specimens were fixed in 4 % paraformaldehyde for 72 h after harvest. All samples were decalcified in 10 % EDTA with PBS at room temperature for 4 weeks. The samples were dehydrated through a graded ethanol series, embedded in paraffin wax, and sectioned at 3 μm parallel to the longitudinal axis of the bone tunnel. Haematoxylin and eosin (H&E) and Russell–Movat pentachrome staining were performed to evaluate tendon–bone healing for conventional light microscopy.

### Radiology and biomechanical testing

The specimens for radiology and biomechanical testing were frozen at −80 °C immediately after harvest. After thawing overnight at 4 °C, the specimens from each group were scanned using a micro-computed tomography (CT) imaging system with a 36-μm isotropic voxel resolution under a 60-kV scanning voltage (Skyscan1176; BRUKER, Antwerp, Belgium).

**Fig. 2:**
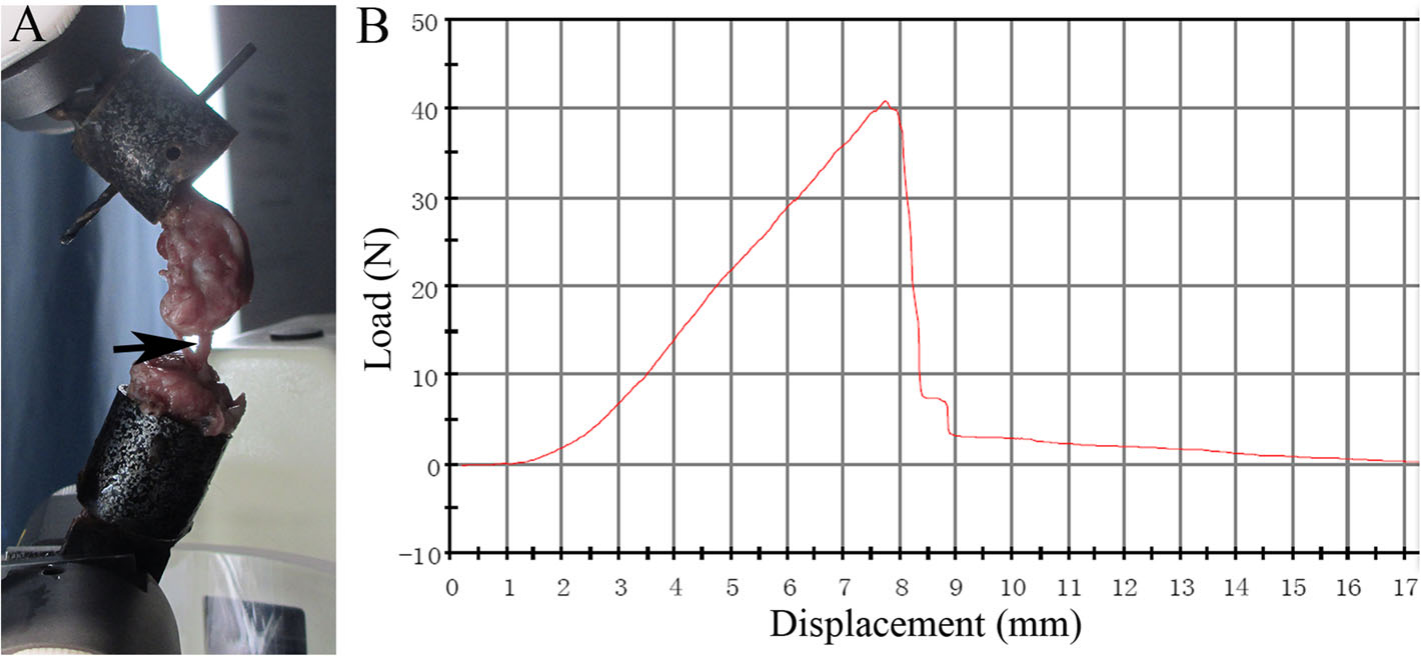
**a** The femoral–graft–tibial complex was firmly fixed on the Instron machine to perform the mechanical test (*black arrow* points to the intra-articular graft). **b** Representative load-deformation curve obtained by the biomechanical test

Biomechanical testing was performed immediately after the scan. All soft tissue except the graft was removed to create a femoral–ACL graft–tibial complex. The femur and tibia were fixed at 45° flexion in an Instron 553A material testing system (Instron, Norwood, MA, USA; Fig. 2a). The test was performed by increasing the tensile load continuously at a speed of 20 mm/min. The failure load (N) was recorded by the load-deformation curve, and stiffness (N/mm) was calculated from the slope of the linear part of the load-deformation curve (Fig. 2b).

### Statistical analysis

All values are expressed as means ± standard deviation, and the statistical analysis was performed using SPSS software (ver. 16.0; SPSS Inc., Chicago, IL, USA). Differences between groups were detected using one-way analysis of variance followed by Scheffe’s multiple comparison test. A *p* value <0.05 was considered significant.

## Results

### Identification of BMSCs

**Fig. 3:**
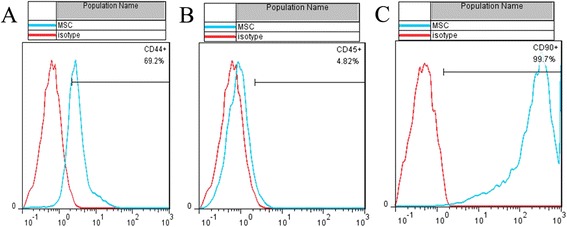
Flow cytometry analysis of passage 3 cell surface markers: 69.2, 4.82, and 99.7 % positive cells for CD44 (**a**), CD45 (**b**), and CD90 (**c**), respectively

**Fig. 4:**
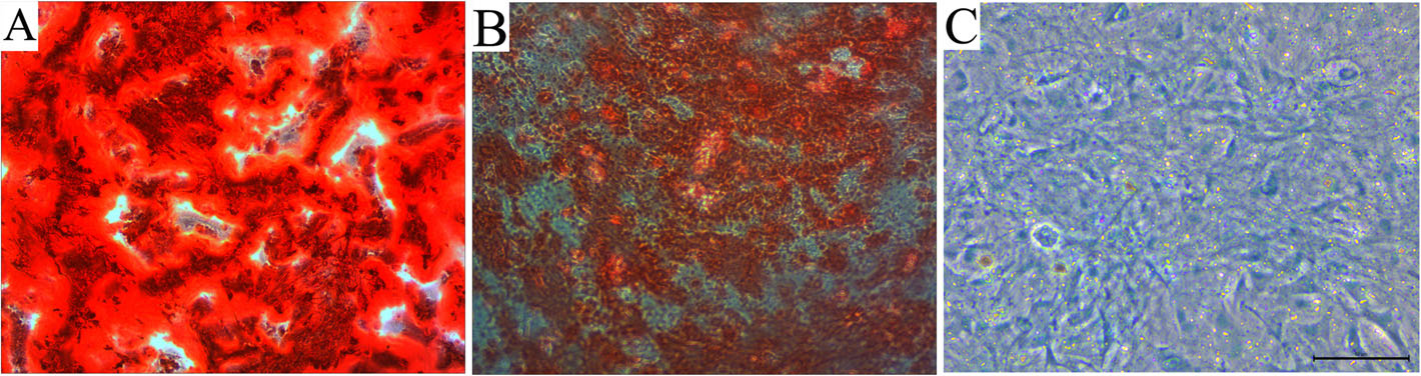
Alizarin red (**a**), oil red O (**b**), and alcian blue staining (**c**) to detect the differentiating ability of bone marrow mesenchymal stem cells (BMSCs) relative to the bone, fat, and cartilage, respectively. Scale bar, 50 μm

A total of 69.2 and 99.7 % of the passage 3 cultured cells expressed CD44 and CD90, respectively, whereas only 4.82 % expressed CD45 and were identified as BMSCs (Fig. 3). Following 3 weeks of culture in osteogenesis induction medium followed by alizarin red staining, obvious mineralised nodules were observed under the microscope (Fig. 4a). Oil red O staining revealed many lipid droplets (Fig. 4b), and the BMSC cytoplasm was stained green by alcian blue after 3 weeks of chondrogenic induction (Fig. 4c).

### Transcription levels of osteogenic genes

**Fig. 5:**
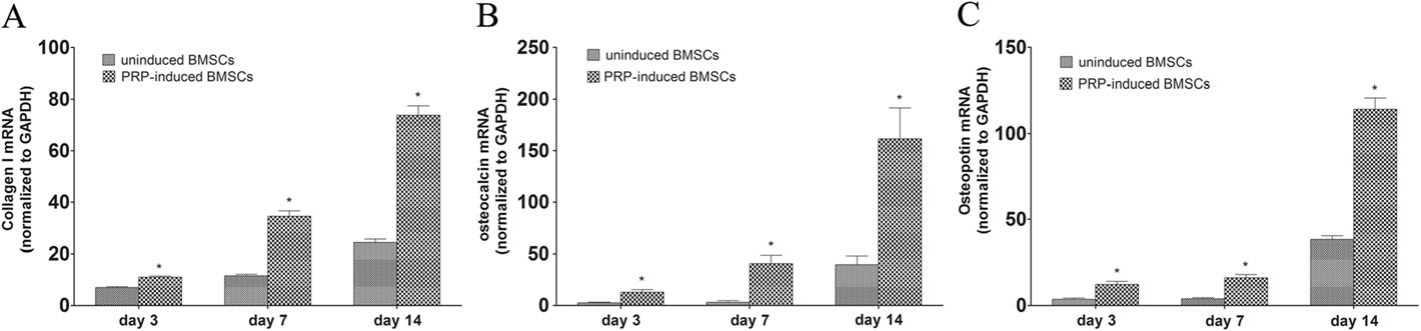
Reverse transcription–polymerase chain reaction evaluation of osteogenic gene mRNA expression. *Asterisk* indicates significant difference (*p <* 0.05) compared with gene expression levels of BMSCs co-cultured with or without platelet-rich plasma (PRP) **a** collagen I; **b** osteocalcin (OCN); **c** osteopontin (OPN)

The transcription of osteogenic genes, including collagen I, osteocalcin, and osteopontin, was evaluated by real-time quantitative reverse transcription–polymerase chain reaction (RT-PCR) analysis. Collagen I, osteocalcin, and osteopontin messenger RNA (mRNA) levels in BMSCs increased gradually after co-culture with PRP. The levels of the three osteogenic genes were significantly higher than those in uninduced BMSCs at all time points (*p <* 0.05, Fig. 5).

### Histological observations

**Fig. 6:**
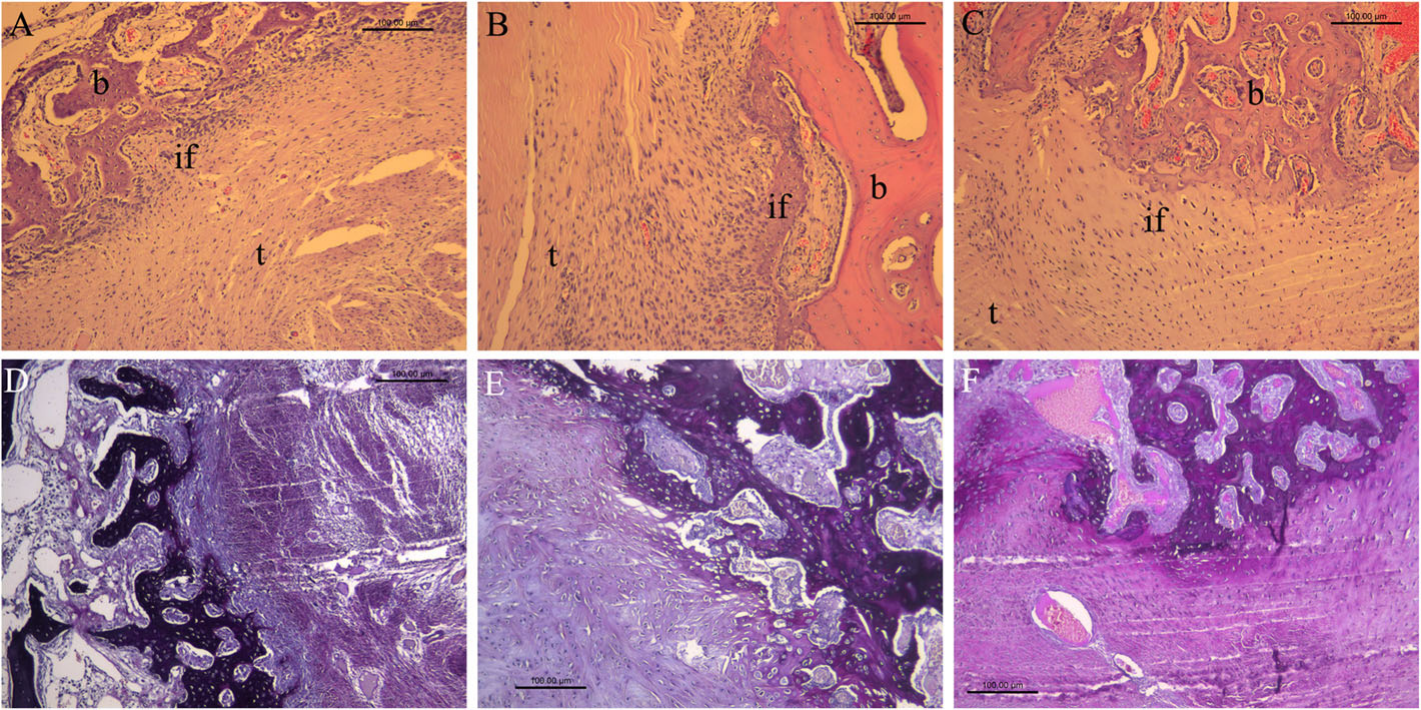
Histological observations of the tendon–bone interface in the control (**a**, **d**), PRP (**b**, **e**), and BMSC + PRP groups (**c**, **f**) by haematoxylin and eosin (H&E) (**a–c**) and Russell–Movat pentachrome staining (**d–f**). Magnification, ×100; scale bar, 100 μm. *t* tendon graft; *b* bone; *if* interface

Organised fibrous tissue and some new bone containing chondrocytes were observed at the tendon–bone interface in the control group 8 weeks postoperatively (Fig. 6a, d). Aligned connective tissue, newly formed woven bone, and cartilage were observed at the tendon–bone interface in the PRP group (Fig. 6b, e). A more mature interface with aligned chondrocytes was observed in the BMSC + PRP group, but the fibrous connective tissue at the tendon–bone interface was unclear. A more aligned and layered cartilage zone was observed, which incorporated adjacent bone and tendon (Fig. 6c, f).

### Micro-CT scan

**Fig. 7:**
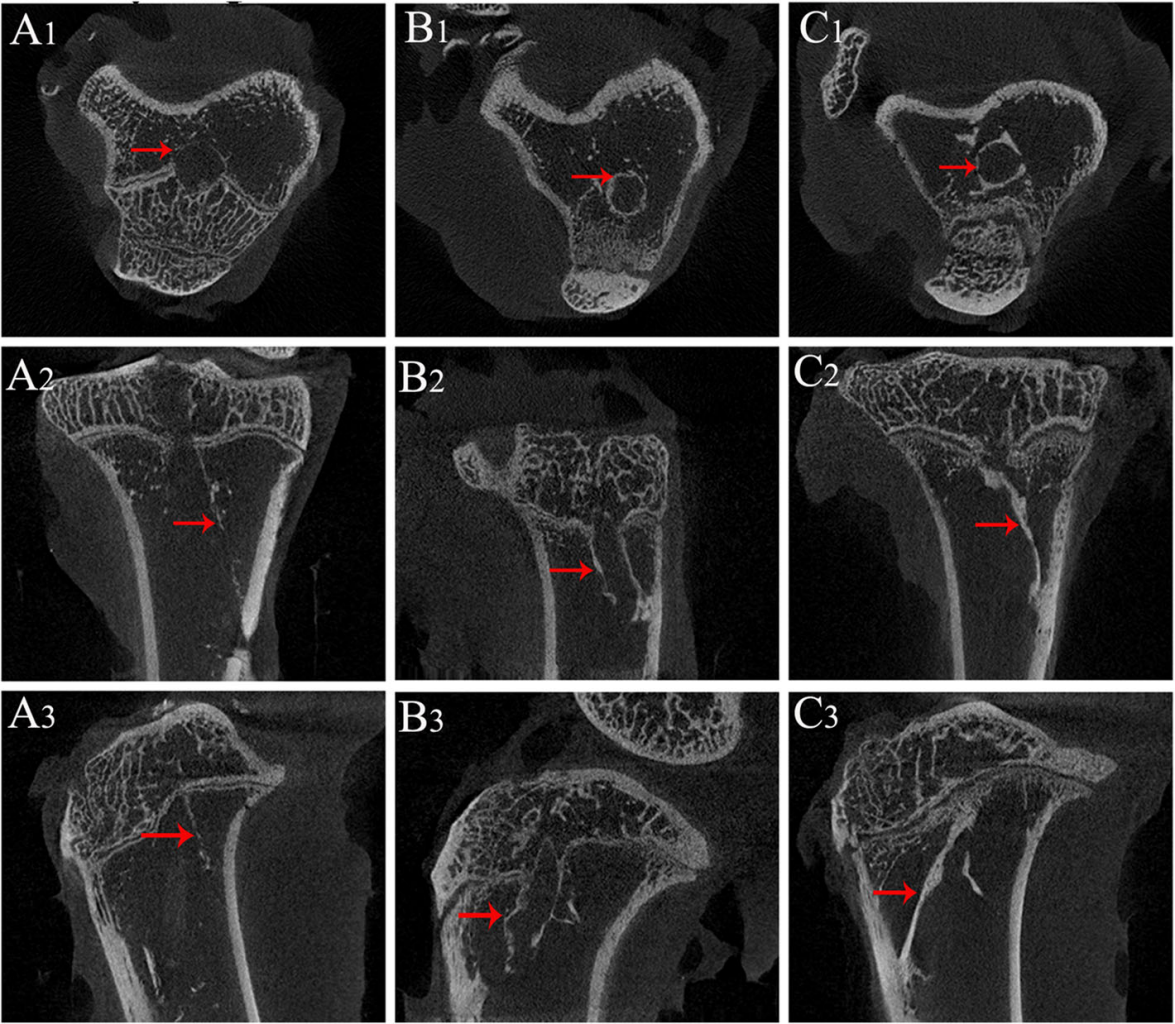
Representative transverse (**a1**, **b1**, and **c1**), coronal (**a2**, **b2**, and **c2**), and sagittal (**A3**, **b3**, and **c3**) section micro-computed tomography images in the three groups. No obvious mineralised tissue had formed in the tibial bone tunnels in the control group (**a1**–**3**). Clear mineralised tissue had formed at the tendon–bone interface in the PRP (**b1**–**3**) and BMSC + PRP groups (**c1**–**3**). The *red arrows* point to the newly formed mineralized tissue around the wall of the tibial bone tunnel

The transverse, coronal, and sagittal section images of the tibial bone tunnel were reconstructed with high-resolution micro-CT. Newly formed mineralised tissue was evident along the entire length of the bone tunnel by screening slices of each sample. The control micro-CT images showed no obvious mineralised tissue in the tibial bone tunnels 8 weeks postoperatively (Fig. 7a1–3). Obvious signals were detected in the bone tunnels of the PRP (Fig. 7b1–3) and BMSC + PRP groups (Fig. 7c1–3), indicating mineralised tissue formation at the tendon–bone interface. A stronger signal was observed in the BMSC + PRP group than in the PRP group, indicative of more mineralised tissue formation.

### Biomechanical testing

**Fig. 8:**
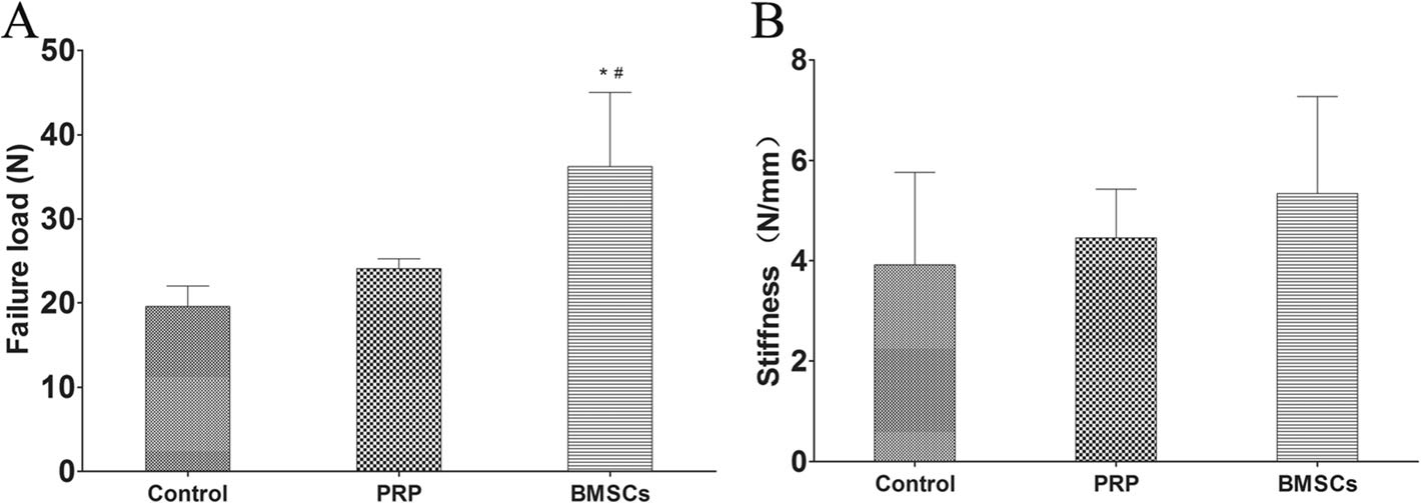
Biomechanical test results: failure load (**a**) and stiffness (**b**). *p < 0.05 vs. control group; ^#^ *p <* 0.05 vs. the PRP group

The failure load (36.22 ± 8.77 N, *n* = 5) was significantly greater in the BMSC + PRP group 8 weeks postoperatively than in the control (19.56 ± 2.45 N, *n* = 5; *p* = 0.001) and PRP groups (24.08 ± 1.16 N, *n* = 5; *p* = 0.012). No difference was observed between the control and PRP groups (*p* = 0.429; Fig. 8a). No difference in stiffness was observed among the groups (Fig. 8b) (Additional file 1).

## Discussion

This study demonstrated that the combination of PRP and BMSCs enhanced tendon–bone healing in a rabbit model of ACL reconstruction. The aligned and layered cartilage zone revealed that directly inserting the tissue resembled the histology of inserting a native ACL. More mineralised tissue had formed, as detected by micro-CT, and a larger failure load was observed in the BMSC + PRP group, indicative of better osteointegration at the tendon–bone interface. These results indicate that the BMSC + PRP combination is a promising way to promote tendon–bone healing.

BMSCs have the potential for differentiation as multipotent stem cells, but this process is not completely spontaneous. Many studies have shown that some cytokines have stimulatory effects on the differentiation of BMSCs [19–21]. In the present study, collagen I, osteocalcin, and osteopontin mRNA levels increased gradually when cells were co-cultured with PRP for 14 days. Activated platelets release TGF-β, PDGF, VEGF, and epidermal growth factor, which promote wound healing [22]. de Mos et al. found that PRP enhanced human tendon cell proliferation and total collagen production in an in vitro study [23].

Tendon–bone healing is slow because the reconstructed tendon graft in the bone tunnel is separated from a vascular supply and the bone is lost at the injury site. During healing, the structure and composition of the native direct tendon–bone interface is not reformed, but a mechanically and structurally inferior interface forms [24]. Previous studies have demonstrated that osteoinductive agents accelerate osteointegration to the tendon graft, improving tendon–bone healing and the mechanical properties [25–27]. Rodeo et al. reported a narrower interface zone in bone morphogenetic protein-treated specimens, indicative of better tendon–bone healing [26]. In the present study, we found that PRP promoted BMSC osteodifferentiation in vitro, and the combination of the two agents promoted tendon–bone healing in vivo.

Successful ACL reconstruction requires solid tendon–bone healing. Direct and indirect insertion are the two types of tendon–bone insertion. Direct insertion, which serves to transmit tensile forces, comprises four typically distinct transition zones, of the bone, mineralised cartilage, fibrocartilage, and ligament. Indirect insertion includes the bone, Sharpey’s fibres (which anchor the soft tissue to the bone), and ligament. The present study showed that PRP and BMSCs histologically promoted bone ingrowth into the tendon–bone interface. An incompletely mature chondral tendon–bone interface was observed in the BMSC + PRP group by H&E and Russell–Movat pentachrome staining.

The walls of the bone tunnel in the PRP and BMSC + PRP groups showed more new bone formation than those of the control group. The newly formed bone represents a direct bonding area between the tendon and bone and prevents the knee instability associated with enlarging the bone tunnel. Enlargement of the bone tunnel at the articular end due to bone resorption is a common problem after ACL reconstruction [28]. New bone formation in the PRP and BMSC + PRP groups could be effective for long-term knee function by preventing the instability associated with bone tunnel enlargement.

The failure load in the BMSC + PRP group was significantly higher than that in the other two groups at 8 weeks postoperatively, but no difference was observed between the PRP and control groups. Tendon–bone healing after ACL reconstruction encompasses an orderly transition of necrosis of graft cells and ingrowth of host cells [13]. The cell types that initiate and regulate tendon–bone healing have not been positively identified until now [4]. Kobayashi et al. reported that if graft cells do not survive the first 2 weeks after ACL reconstruction, the graft will undergo necrosis [13]. It seems that the host cells from the surrounding bone marrow in the bone tunnel, which contains BMSCs and other preosteoblasts, contribute to tendon–bone interface repair [13, 14]. In our study, significant differences in mechanical properties were observed between the BMSC + PRP and the other two groups, indicating that the seeded cells contribute to the tendon–bone healing process.

MSCs have become the gold standard for cellular therapies in musculoskeletal diseases because of their ease of expansion and capability of differentiating into chondrocytes, tenocytes, and osteocytes [29–31]. Several studies have explored the effects of tendon–bone healing with MSCs. Ouyang et al. applied BMSCs at the tendon–bone interface when fixing the hallucis longus tendon in a calcaneal bone tunnel. Only 50 % of the tendon–bone interface contained fibrocartilage, although the BMSC group promoted collagen II staining [32]. Ju et al. explored the effect and mechanism of the implantation of BMSCs on tendon–bone healing in rats. The MSC group had a higher percentage of oblique fibres relative to the total interface area compared with controls [33]. Gulotta et al. reported that the addition of BMSCs to the tendon–bone interface did not improve the composition, structure, or strength of the tendon–bone attachment site [34]. It is possible that the tendon–bone interface lacks the molecular and/or cellular signals necessary for inducing the transplanted cells to appropriate differentiation, implying that MSC-based strategies should be combined with appropriate differentiation and growth factors. In the present study, BMSCs serve as the material for regeneration, while PRP serves as the impetus to promote tendon–bone healing.

A limitation of this study was that the BMSCs were not labelled and tracked. Fan et al. labelled BMSCs with green fluorescence protein (GFP) to examine cell viability on scaffolds implanted into a rabbit knee joint for ACL reconstruction. Their results revealed GFP-positive scaffolds 4 weeks postoperatively, indicating that the BMSCs were viable [35]. Further studies are required to precisely track the fate of implanted BMSCs. Another limitation of this study was that the failure load was tested only under a static condition at 45° of flexion. The biomechanical test was limited by our sample size and our Instron machine, which only tested tensile strength in a straight line.

## Conclusions

PRP significantly stimulated osteogenic differentiation in BMSCs. The combination of PRP and BMSCs enhanced tendon–bone healing in a rabbit model of ACL reconstruction, demonstrating its potential for clinical use.

## Additional file

Additional file 1: Results of biomechanical test. (PDF 94 kb)

## Abbreviations

ACL: Anterior cruciate ligament
BMSCs: Bone marrow mesenchymal stem cells
PRP: Platelet-rich plasma
B-PT-B: Bone–patellar tendon–bone
PDGF: Platelet-derived growth factor
PDEGF: Platelet-derived epidermal growth factor
TGF: Transforming growth factor
VEGF: Vascular endothelial growth factor
DMEM: Dulbecco’s modified Eagle medium
EDTA: Ethylenediaminetetraacetic acid
H&E: Haematoxylin and eosin
Micro-CT: Micro-computed tomography
RT-PCR: Real-time quantitative reverse transcription–polymerase chain reaction
GFP: Green fluorescence protein
