# Supplementary material for: Combination of platelet-rich plasma and bone marrow mesenchymal stem cells enhances tendon–bone healing in a rabbit model of anterior cruciate ligament reconstruction
Source: J Orthop Surg Res. 2016 Sep 7;11(1):96. doi: 10.1186/s13018-016-0433-7 (PMC5015347; doi:10.1186/s13018-016-0433-7)
Supplement: Additional file 1: — Results of biomechanical test. (PDF 94 kb) [file 13018_2016_433_MOESM1_ESM.pdf]

Results of biomechanical test

| group | | Failure load (N) | Deformation (mm) | Stiffness (N/mm) |
|---|---|---|---|---|
| control | 1 | 15.74 | 8.02 | 1.96 |
| | 2 | 18.74 | 4 | 4.69 |
| | 3 | 20.82 | 3.1 | 6.72 |
| | 4 | 20.4 | 6.95 | 2.94 |
| | 5 | 22.11 | 6.72 | 3.29 |
| PRP | 1 | 24.69 | 4.4 | 5.61 |
| | 2 | 23.07 | 5 | 4.61 |
| | 3 | 24.83 | 8.3 | 2.99 |
| | 4 | 22.61 | 4.62 | 4.89 |
| | 5 | 25.21 | 6 | 4.2 |
| BMSCs+PRP | 1 | 30.81 | 4.69 | 6.57 |
| | 2 | 31.62 | 14.15 | 2.23 |
| | 3 | 37.98 | 6.91 | 5.5 |
| | 4 | 50.84 | 7.02 | 7.24 |
| | 5 | 29.87 | 5.77 | 5.18 |
